# Health promotion at work: assessment of lifestyles of administrative assistants in a hospital

**DOI:** 10.1590/1980-220X-REEUSP-2022-0198en

**Published:** 2022-09-19

**Authors:** Paula Alexandra de Melo Farias, Sara Raquel Ferreira Raposo, Helder José Alves da Rocha Pereira

**Affiliations:** 1 Hospital do Divino Espírito Santo de Ponta Delgada, Serviço de Saúde Ocupacional, Açores, Portugal.; 2 Hospital do Divino Espírito Santo de Ponta Delgada, Açores, Portugal.; 3 Universidade dos Açores, Escola Superior de Saúde, Departamento de Enfermagem, Saúde da Família, e Comunidade, Açores, Portugal.

**Keywords:** Occupational Health, Health Promotion, Healthy Lifestyle, Salud Laboral, Promoción de la Salud, Estilo de Vida Saludable, Saúde do Trabalhador, Promoção da Saúde, Estilo de Vida Saudável

## Abstract

**Objective:**

To characterize the lifestyles of administrative assistants in a hospital, in order to define health promotion strategies in the workplace.

**Method:**

A quantitative, descriptive, cross-sectional study, carried out with administrative assistants (N = 167) of a medium-sized Portuguese hospital. The data were collected through a questionnaire (QEV&PS-SO) for sociodemographic characterization and analysis of health determinants related to lifestyles.

**Results:**

It was observed that 54% of the sample did not practice physical exercise, 52% had four or fewer meals a day, 29% were smokers, 51% had insomnia, and 45% had no health surveillance, as well as 51.5% were overweight or obese and 63% had an increased or very increased risk of developing metabolic complications. No significant differences were identified in relation to weight, physical exercise or stress according to sex or age.

**Conclusion:**

The results support the need to develop intervention programs with strategies aimed at promoting healthy lifestyles among workers in health institutions, to be comprehensively integrated within the scope of occupational health.

## RESUMO

**Objetivo:**

Caracterizar os estilos de vida dos assistentes administrativos de um hospital, para definição de estratégias de promoção de saúde no local de trabalho.

**Método:**

Estudo quantitativo, descritivo, de caráter transversal, realizado junto a assistentes administrativos (N = 167) de um hospital português de média dimensão. Os dados foram coletados através de um questionário (QEV&PS-SO), para caracterização sociodemográfica e análise dos determinantes de saúde relacionados com estilos de vida.

**Resultados:**

Observou-se que 54% da amostra não praticava exercício físico, 52% fazia quatro ou menos refeições por dia, 29% apresentava hábitos tabágicos, 51% tinha insônia e 45% não fazia vigilância de saúde, assim como 51,5% apresentava excesso de peso ou obesidade e 63% apresentava risco aumentado ou muito aumentado para desenvolvimento de complicações metabólicas. Não se identificaram diferenças significativas em relação ao peso, prática de exercício físico ou estresse em função do sexo ou idade.

**Conclusão:**

Os resultados sustentam a necessidade do desenvolvimento de programas de intervenção com estratégias dirigidas à promoção de estilos de vida saudáveis junto aos trabalhadores das instituições de saúde, a serem integradas, de forma compreensiva, no âmbito da saúde ocupacional.

## RESUMEN

**Objetivo:**

Caracterizar los estilos de vida de los auxiliares administrativos de un hospital, para definir estrategias de promoción de la salud en el trabajo.

**Método:**

Estudio cuantitativo, descriptivo, transversal, realizado con auxiliares administrativos (N = 167) de un hospital portugués de tamaño medio. Los datos fueron recolectados a través de un cuestionario (QEV&PS-SO), para la caracterización sociodemográfica y el análisis de los determinantes de la salud relacionados con los estilos de vida.

**Resultados:**

Se observó que el 54% de la muestra no practicaba ejercicio físico, el 52% hacía cuatro o menos comidas al día, el 29% era fumador, el 51% tenía insomnio, el 45% no tenía vigilancia de salud, así como el 51,5% tenía sobrepeso u obesidad y el 63% tenía un riesgo aumentado o muy aumentado de desarrollar complicaciones metabólicas. No se identificaron diferencias significativas en relación con el peso, el ejercicio físico o el estrés según el sexo o la edad.

**Conclusión:**

Los resultados respaldan la necesidad de desarrollar programas de intervención con estrategias dirigidas a la promoción de estilos de vida saludable entre los trabajadores de las instituciones de salud, para ser integradas integralmente en el ámbito de la salud ocupacional.

## INTRODUCTION

Occupational health has evolved from an activity oriented towards the assessment of occupational risk and protection against occupational disease to a holistic and comprehensive approach that takes into account workers’ physical, mental and social well-being. The right to a safe and healthy work environment is fundamental, and special attention is needed to implement preventive strategies that guarantee and promote worker safety and health^(1)^.

Luxembourg Declaration on Health Promotion in the Workplace in the European Union, as early as 1997, aimed to achieve the goal of healthy people in healthy organizations^(1)^. More recently, in the European Union, the strategic framework for health and safety at work 2021–2027 reinforces this perspective, arguing that encouraging the adoption of healthy lifestyles in the workplace can reduce absenteeism and non-communicable diseases, such as cancer, obesity, cardiovascular disease and diabetes^(2)^.

In Portugal, the Portuguese National Occupational Health Plan (PNSOC – Extension 2018/2020) gives visibility to a set of principles for the organization of care, with a view to supporting workers’ health such as: (i) the prevention of professional risks through the fight against risk factors; (ii) protection of workers’ health and well-being through early diagnosis and treatment of work-related illnesses; and (iii) the promotion of healthy work environments that, in addition to safe working conditions, offer opportunities for improving individual health and reinforcing healthy practices and lifestyles^(3)^. This perspective values the integration of strategies to promote workers’ individual health and acquisition/reinforcement of healthy lifestyles within the scope of the attributions and responsibilities of occupational health services.

The way in which each person manages their own health capital throughout their lives, through individual options corresponding to the so-called “lifestyle”, is a crucial issue that is at the genesis of individual and collective health^(4)^. The World Health Organization (WHO) reiterates that health promotion should be more encouraged in the workplace, primarily by encouraging the adoption of healthy eating, physical activity and mental and family health promotion^(5,6,7,8,9)^. An unhealthy diet, smoking, sedentary lifestyle and increased alcohol consumption significantly increase the risk of cardiovascular disease, cancer occurrence, loss of healthy life years, leading to premature mortality^(5)^.

The estimated number of work-related illnesses far exceeds the number of accidents at work and deaths^(3)^. At the national level, chronic pain, particularly low back and cervical pain, are the diseases most frequently reported by the population aged 15 or over: 2.9 and 2.1 million people, respectively, i.e., 32.9% and 24.1%^(10)^. In addition to these problems, psychosocial risks (stress, depression, anxiety and burnout situations) are an increasingly present reality in work environments, given the enormous pressure to respond to the contemporary work environment and the current pandemic situation demands^(11)^. Work-related illnesses, injuries and deaths result in high economic costs for individuals, employers, governments and society. Negative effects may include costly early retirement, loss of skilled personnel, absenteeism, presenteeism, and high medical costs and insurance premiums^(12,13)^.

The implementation and maintenance of health promotion actions and programs in the workplace lead to positive results for the organization, as they promote healthy lifestyles, prevent diseases, contribute to occupational health and improve the organizational climate^(14)^.

However, few studies have been developed in the area of promoting healthy lifestyles in the work environments of institutions, particularly those related to health care provision. Despite some published studies aimed at nurses and physicians, there is very little about the professionals of these institutions who play an administrative role. Administrative assistants, along with other professionals, are fundamental elements in the multidisciplinary team of any health unit, as they are the intermediate between the public and the institution’s professionals. Taking into account the nature of their functions, they are often exposed to ergonomic risks (repetitive and/or prolonged postures, long periods of time in a sitting position, among others) and psychosocial risks (multiple pressures from various professional groups and the public they serve, intensity of work, accumulation of functions). In this context, it is important that health institutions, within the scope of occupational health intervention, know the health status of their professionals, in order to design intervention strategies that promote healthy lifestyles and consequent health gains.

In this regard, this study aimed to characterize the lifestyles of administrative assistants in a hospital, with a view to defining strategies for health promotion in the workplace.

## METHOD

### Study Design

This is a quantitative, descriptive and cross-sectional study.

### Population and Sample

The study population was considered to be the set of administrative assistants of the selected hospital, regardless of employment relationship with the institution. The non-probabilistic sample was used for convenience.

### Local

Medium-sized Portuguese hospital (400 beds) with differentiated offer of specialized care.

### Selection Criteria

The sample selection criteria were (i) to perform functions as administrative assistant at the institution and (ii) to demonstrate interest in participating in the study. Of all the institution’s administrative assistants (204), it was possible to contact 179, and 167 (82%) agreed to voluntarily participate in the study.

### Data Collection

To collect the information, the “Questionnaire on Lifestyles and Health Promotion in Occupational Health” (QEV&PS-SO) was prepared. The first version of the QEV&PS-SO was submitted to content validity by experts in the areas of occupational health, community nursing and sociology. Judges’ assessments were integrated into a second version of the information collection instrument, which was submitted to a pre-test with health professionals belonging to different professional categories. With the pre-test, we intend to verify the clarity and usefulness of the questions, the question order adequacy, response time and possible constraints in the questionnaire application^(15)^. The final version of the questionnaire comprises two distinct parts, the first being aimed at characterizing the population (age, sex, household composition, residence, education level and working conditions) and the second referring to the different dimensions under study: (i) health status; (ii) lifestyles (physical activity, eating habits, tobacco consumption, alcohol consumption, sleep); (iii) stress; and (iv) access to health services. The collection of information, using the interview carried out by the project researchers, lasted approximately 30 minutes, and took place in January 2017, during the work activity of administrative assistants. At the same time, anthropometric measurements (weight, height and abdominal perimeter) were collected and recorded.

### Data Analysis and Treatment

The collected data were analyzed using descriptive statistics (absolute and relative frequencies, means and standard deviation) and inferential (Mann-Whitney and Kruskal-Wallis non-parametric chi-square and non-parametric independence tests), using the SPSS program (version 28.0).

### Ethical Aspects

Prior to data collection, a favorable opinion was obtained from the Ethics Committee of the *Universidade dos Açores* (Opinion 1/2017). Access to the research site was granted by the hospital’s board of directors. Informed consent was obtained from all participants.

## RESULTS

Through the data collection instrument completion, 167 administrative assistants (82% of the administrative staff) agreed to participate in the study, 135 (81%) being female, aged between 22 and 63 years. Participants were mostly married (64%), with complete secondary education (65%) and residing in the geographic area of the hospital (80%). Of the respondents, 60% had been working for at least 16 years in the professional category, with the most expressive employment relationship being an Indefinite Term Contract (75%). with a weekly workload of 35 hours (66%) and predominantly daytime (87%).

Regarding anthropometric measurements, information regarding body weight, height and abdominal perimeter (AP) was collected. Obtaining these data allowed calculating the Body Mass Index (BMI) of each participants. The most relevant data for this assessment are presented in Table 1.

**Table 1.** Categories of Body Mass Index by age group – Ponta Delgada, Portugal, 2017.

			Age group				Total
			<30	30–39	40–49	>/=50	
BMI cat
	Low weight	n	0	1	1	2	4
		% in cat. BMI	0.0%	25.0%	25.0%	50.0%	100.0%
		% in age group	0.0%	1.9%	1.7%	4.5%	2.4%
		% of total	0.0%	0.6%	0.6%	1.2%	2.4%
	Healthy weight	n	7	27	27	16	77
		% in cat. BMI	9.1%	35.1%	35.1%	20.8%	100.0%
		% in age group	63.6%	51.9%	45.0%	36.4%	46.1%
		% of total	4.2%	16.2%	16.2%	9.6%	46.1%
	Overweight	n	3	13	20	16	52
		% in cat. BMI	5.8%	25.0%	38.5%	30.8%	100.0%
		% in age group	27.3%	25.0%	33.3%	36.4%	31.1%
		% of total	1.8%	7.8%	12.0%	9.6%	31.1%
	Obesity	n	1	11	12	10	34
		% in cat. BMI	2.9%	32.4%	35.3%	29.4%	100.0%
		% in age group	9.1%	21.2%	20.0%	22.7%	20.4%
		% of total	0.6%	6.6%	7.2%	6.0%	20.4%
	Total	n	11	52	60	44	167
		% in cat. BMI	6.6%	31.1%	35.9%	26.3%	100.0%
		% in age group	100.0%	100.0%	100.0%	100.0%	100.0%
		% of total	6.6%	31.1%	35.9%	26.3%	100.0%

BMI – Body Mass Index.

With regard to BMI, it was found that 51.5% (n = 86) was within the scope of overweight or obesity categories. In the sample studied, BMI does not vary according to sex (U = 1730.50; p = 0.60) or age [(χ^2^ = 3.378; p = 0.760). For this calculation, the categories of BMI were considered, <24.9; 25–29.9 and ≥30, in order to guarantee the conditions of execution of the statistical test].

It was also observed that 56% (n = 18) of male participants and 66% (n = 88) of female participants had an increased or very increased risk of developing metabolic complications, since they had AP equal to or greater than 94 cm and 80 cm, respectively, which corresponds to 63% (n = 106) of the total sample.

With regard to lifestyle assessment, data collection related to physical activity, eating habits and sleep and resting habits was privileged.

Participants’ physical activity included tendentially sedentary habits, and 54% did not practice physical activity (n = 89), with no significant differences according to age group (χ^2^ = 3.757; p = 0.289) or sex (U = 1988.50; p= 0.420).

**Chart 1:**
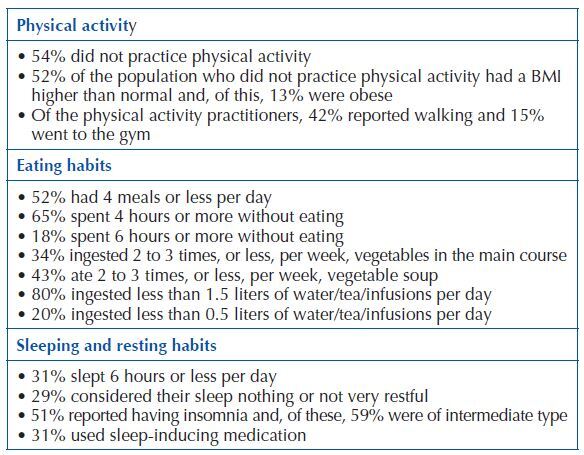
Synthesis of data related to lifestyles – Ponta Delgada, Portugal, 2017.

For the study of eating habits, in addition to the number of meals eaten daily, the interval time between meals and the type of food usually eaten (including vegetables, soup and liquids) were questioned. Most participants had a limited number of meals, with prolonged intervals between them throughout the day. The intake of vegetables in the main course and weekly soup was reduced, as was the percentage of participants who ingested more than 1.5 liters of water/tea/infusions per day. Regarding sleep and rest, participants reported sleeping an average of 6.87 hours a day (±1.248), with a minimum of 3 hours and a maximum of 10 hours (Chart 1).

Crossing the BMI data with the intensity of physical activity practiced revealed that 52% (n = 46) of the population that did not practice physical activity had a BMI higher than normal and, of these, 13% (n = 11) were obese. It was also observed that 24% (n = 6) of those who practiced intense physical activity were overweight, and 16% (n = 4) were obese.

Regarding tobacco consumption, it was verbalized by 29% of the population, with an average daily consumption of 11 (±5.866) cigarettes per day, ranging between 2 and 30. Of the smoking participants, 42% had been smoking for more than 20 years and 48% assumed they had never tried to quit smoking.

Alcohol consumption, despite having been reported by 58% (n = 97) of participants, only in 5% of cases (n = 5) it was observed that this consumption was daily.

**Chart 2:**
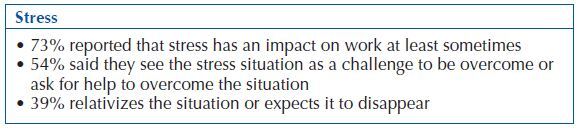
Synthesis of data related to workers’ perception of stress – Ponta Delgada, Portugal, 2017.

Regarding stress, 83% (n = 138) of the respondent population reported feeling at least under stress sometimes in the workplace. The most verbalized reasons as causing stress were excess functions (34%) and noise in the workplace (34%) (Chart 2). In this variable, no significant differences were observed according to age group (H = 6.304; p = 0.098) or sex (U = 1721.50; p = 0.059).

With regard to access to health services, the following indicators were addressed: adequate demand for health services, ease of access, frequency of appeal and level of satisfaction. In the same theme, the perception of the existence of an OHS (occupational health service), knowledge/use of available resources, frequency of use and the importance of the existence of the service in the hospital were studied.

**Chart 3:**
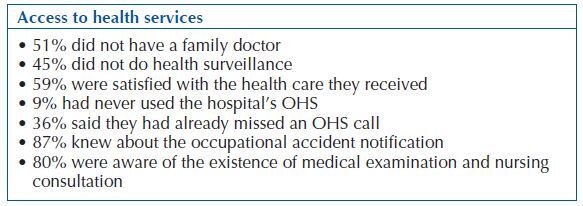
Synthesis of data related to access to health services – Ponta Delgada, Portugal, 2017.

During the data collection period, the population studied without a family doctor or with inadequate health surveillance was considerable. The entire population was aware of the existence of OHS in the hospital, and 95% considered it important or very important (Chart 3).

OHS – occupational health service.

## DISCUSSION

Working conditions and environment condition workers’ health and well-being^(1)^. A successful organization is based on healthy and empowered workers^(16)^, providing the basis for a strong and resilient economy and society^(2)^.

In the professional group of administrative assistants under study, more than half of participants were overweight (39% overweight + 13% obesity) and/or at increased or greatly increased risk for the development of metabolic complications. These results are comparable with data from the general population in Portugal. In 2019, more than half of the Portuguese population aged 18 or over (53.6%) was overweight or obese, i.e., BMI of 25 or more kg/m^2^. Obesity (30 or more kg/m^2^) reached 1.5 million people aged 18 or over (16.9%), with women being more affected than men (17.4% and 16.4%, respectively)^(17)^. In turn, the population living in the Autonomous Region of the Azores recorded the highest proportions of people with a BMI classified as obesity, especially the female population (25.3%). Given the costs that have consequences on the population’s health and quality of life, as well as the difficulty of its treatment^(18)^, obesity is one of the most important public health problems in Portugal, which requires a concerted strategy that includes the promotion of healthy eating habits and a more active life^(6,7,19)^. Lifestyle modification is not a short-term effort, and maintaining a healthy weight requires sustained changes in the pattern of physical activity and individual eating habits^(20,21)^.

In the study population, maladjusted eating habits and a low percentage of regular physical activity practitioners were identified.

With regard to eating habits, among other determinants, there was a deficit in the weekly intake of vegetables and greens and the occurrence of extended periods of interval between meals. Excessive salt consumption, insufficient consumption of fruit, vegetables, whole grains and oleaginous fruits are among the main inappropriate eating behaviors of Portuguese individuals^(22)^. Inadequate eating habits constitute the third risk factor for loss of healthy life years^(22)^. Adults of working age spend a large part of their day at work, which is why it is considered pertinent to provide information on healthy eating and the creation of conditions that allow workers to apply good food practices in work contexts, namely by providing healthy food in bars and cafeterias and sufficient time to eat meals^(23)^.

In turn, physical activity is an important therapeutic adjuvant, by increasing longevity and contributing to the delay of the progression of several chronic non-communicable diseases, such as cardiovascular, oncological, pulmonary, metabolic, psychiatric, neurological and musculoskeletal pathologies^(6,7)^.

A healthy lifestyle inevitably translates into the regular practice of exercise, whether programmed or not, and simultaneously, in the opposite of sedentary behaviors^(4,6,7)^. The World Health Organization launched at the end of 2020 the new global recommendations for physical activity and sedentary behavior^(4)^. In light of new scientific evidence, all physical activity, regardless of the consecutive duration of each practice period, has a positive impact on health, from walking or climbing stairs to scheduled physical exercise or sports activity. In the workplace context, policies and programs should include the design of spaces that promote incidental physical activity, promotion of active mobility, encouraging an active work culture (e.g., traveling meetings) and providing workers with paid or flexible hours for physical activity, education sessions, etc^(7)^.

Tobacco consumption was identified in 29% of participants. These data prove to be superior both in comparison with the data for the Portuguese population in general (16.8%) and for the Azores Region (23.4%) population^(18)^, constituting an aspect that deserves special attention. According to the latest estimates prepared by the Institute for Health Metrics and Evaluation (IHME), in 2019, more than 13,500 people died in Portugal from diseases attributable to tobacco^(24)^. Tobacco consumption, in the long term, can cause damage to various systems of the body, being the cause or aggravating factor of the most prevalent chronic non-communicable diseases, in particular cancer, respiratory diseases, cardiovascular diseases and diabetes, in addition to other harmful effects on sexual and reproductive health, eye health, oral health and skin aging^(24)^. It is important that workplaces foster awareness strategies for smoking cessation and that workers are aware of the resources available in organizations for this purpose^(25)^.

The self-reported alcohol consumption by participants was considered sporadic and performed essentially in a social context. These data must, however, be interpreted with caution and may be underrepresented, considering the content of the question and the context in which the information was collected.

Sleep disturbances can affect the ability to work. In the study group, it was observed that, on average, administrative assistants reported sleeping 6.87 hours (±1.248), which did not seem to be a problem. However, half of them report having intermediate insomnia and 29% consider sleep to be not at all restful. These complaints are in line with those described by the DGS as the most common and valued by workers in general and for which organizations should be aware, depending on the work contexts^(10)^.

Workers exposed to psychosocial risks may develop stress, negatively affecting the performance of their functions. In the European Union, stress is one of the greatest causes of work-related illnesses^(1)^. Of the population studied, 39% (n = 65) reported feeling under stress frequently or almost always in the workplace. In a similar study using the Job Stress Scale, it was identified that 16.2% of hospital professionals were subjected to high stress exposure, and 11.2% to intermediate exposure^(26)^. These results corroborate the literature, which calls for strengthening the study of psychosocial risks in workers’ health, suggesting the proper monitoring and implementation of stress prevention strategies among health professionals^(1)^.

Regarding access to health services, in the hospital under analysis, more than half of the workers studied did not have a family doctor or had inadequate health surveillance. The success of occupational disease prevention is related to the participation of both the occupational physician and other health professionals, in order to identify, as early as possible, health deviations originating from the professional activity^(1)^.

Health risk assessment of workers is extremely important to legitimize the development of health promotion projects, in order to establish, together with the OHS team, priorities, goals, strategies and the resources necessary to achieve the goals. defined actions^(25)^. The results of this study point to a particular attention in the studied group with regard to combating problems such as obesity, sedentary habits, inadequate eating habits, smoking habits and stress management. Given the characteristics of the functions performed, similar groups of workers may be expected to face similar problems.

In this context, nursing plays a preponderant role, because it is a profession strongly oriented towards health promotion. In Portugal, despite the fact that, since 2018, the Order of Nurses has regulated increased competence in occupational nursing, the role of occupational health nurses in the OHS team still lacks depth and projection. The focus on studies, such as the one presented here (centered on the diagnosis of health needs and oriented towards the design of health promotion projects and programs), gives visibility to the field of action of nurses in this field and reinforces the gains that workers and organizations can benefit from using interventions of this nature.

The implementation of health promotion programs in the workplace implies a continuous commitment by all parties involved, combining the needs of the organization with those of workers, where health and safety services at work play a decisive role^(16,23)^.

Workplaces are privileged spaces for health promotion, with direct effects on workers’ health, resulting in clear benefits for employers^(10)^. In addition to the benefits for workers’ health, at the organizational level, its implementation may provide a better working environment, reduced absenteeism, reduced employee turnover and, in the long term, improved the company’s image, strengthening its competitiveness and increasing productivity^(25)^.

Companies that view the safety and health of their workers as an investment rather than a cost observe a wide range of benefits, assuming that prevention is cost-effective and should be strategically used^(1)^. At the level of society, quality occupational safety and health reduces the burden on general health care and other social expenditure^(2)^.

## CONCLUSION

The present study characterized the lifestyles of administrative assistants in a medium-sized hospital.

The scope of this study falls within the scope of intervention in occupational health, focusing on the promotion of healthy lifestyles among workers, an area that has been little explored by organizations.

The results obtained identify, for the studied group, areas that could constitute priority problems for intervention through the design of health promotion programs and projects, designed to manage the most relevant situations detected (deficit in physical activity, inappropriate eating behaviors, excess weight, sleep quality, among others) and that can significantly compromise or worsen workers’ health status and quality of professional and personal life, with a direct impact on the performance of employing organizations.
